# Cardiorespiratory diseases in an industrialized area: a retrospective population-based cohort study

**DOI:** 10.1186/s12889-023-16925-9

**Published:** 2023-10-18

**Authors:** Elisa Bustaffa, Cristina Mangia, Liliana Cori, Fabrizio Bianchi, Marco Cervino, Fabrizio Minichilli

**Affiliations:** 1 Institute of Clinical Physiology, National Research Council, Via Moruzzi 1, Pisa, 56124 Italy; 2 Institute of Atmospheric Sciences and Climate, National Research Council, Strada Prov.le Lecce-Monteroni Km 1,200, Lecce, 73100 Italy; 3 Institute of Atmospheric Sciences and Climate, National Research Council, Via Gobetti 101, Bologna, 40129 Italy

**Keywords:** Industrial exposure, Cardio-respiratory diseases, Residential cohort study, Mortality, Morbidity, Hazard ratio, Dispersion model

## Abstract

**Background:**

Atmospheric pollution has been recognized as the greatest environmental threat to human health. The population of the Venafro Valley, southern Italy, is exposed to emissions from a Waste-To-Energy (WTE) and a cement plant and potentially also to another WTE located in the neighboring region of Lazio; also, the vehicular atmospheric pollution situation is critical. In order to assess the environmental health risk of residents in eight municipalities of the Venafro Valley, a retrospective residential cohort study during 2006–2019 was carried out.

**Methods:**

Four exposure classes were defined by natural-break method, using a dispersion map of nitrogen dioxides (chosen as proxy of industrial pollution). The association between the industrial pollution and cause-specific mortality/morbidity of the cohort was calculated using the Hazard Ratio (HR) through a multiple time-dependent and sex-specific Cox regression adjusting for age, proximity to main roads and socio-economic deprivation index.

**Results:**

Results showed, for both sexes, mortality and morbidity excesses in the most exposed class for diseases of the circulatory system and some signals for respiratory diseases. Particularly, mortality excesses in both sexes in class 3 for diseases of the circulatory system [men: HR = 1.37 (1.04–1.79); women: HR = 1.27 (1.01–1.60)] and for cerebrovascular diseases [men: HR = 2.50 (1.44–4.35); women: HR = 1.41 (0.92–2.17)] were observed and confirmed by morbidity analyses. Mortality excesses for heart diseases for both sexes [men-class 3: HR = 1.32 (0.93–1.87); men-class 4: HR = 1.95 (0.99–3.85); women-class 3: HR = 1.49 (1.10–2.04)] and for acute respiratory diseases among women [HR = 2.31 (0.67-8.00)] were observed. Morbidity excesses in both sexes for ischemic heart diseases [men-class 3: HR = 1.24 (0.96–1.61); women-class 4: HR = 2.04 (1.04–4.02)] and in class 4 only among men for respiratory diseases [HR = 1.43 (0.88–2.31)] were also found.

**Conclusions:**

The present study provides several not-negligible signals indicating mitigation actions and deserve further investigations. For future studies, the authors recommend enriching the exposure and lifestyle profile using tools such as questionnaires and human biomonitoring.

## Introduction

Atmospheric pollution originates from both natural and anthropogenic sources and has been recognized as the greatest environmental threat to human health globally, on the basis of its significant contribution to the burden of disease. The World Health Organization estimates that around 7 million deaths, mainly from non-communicable diseases, are attributable to the joint effects of environmental and domestic air pollution [1]. Similar global assessments of ambient air pollution alone suggest between 4 million and 9 million deaths per year and hundreds of millions of years of healthy life lost, with the highest attributable disease burden observed in low- and middle-income countries [1–4].

In the many situations where population centres are close to industrial areas, both workers and the general population are exposed to industrial pollution and among the sources of hazardous industrial emissions, waste-to-energy (WTE) and cement plants are often represented.

Since the World Energy Council estimates that global waste production will double from 3 to over 6 million tons per day in the decade from 2015 to 2025 [5], in order to manage this huge mass of Municipal Solid Waste (MSW), WTE has become the most widely used method, being able to reduce the volume of landfills while providing energy [6]. The combustion of MSW is the most consolidated method in the world for energy recovery, accounting for almost 90% of the WTE sector [5]. The combustible component of MSW is known as Refuse-Derived Fuel (RDF) and is used to generate electricity or heat, combustible gases and solids as main recovery products [6]. However, the WTE process involves the combustion of RDF components with consequent potential emissions of persistent organic pollutants such as dioxins [7], but it also emits carbon dioxide (CO_2_), sulfur dioxide (SO_2_), nitrogen oxides (NO_x_) per unit of electricity produced higher than other forms of energy such as natural or renewable gas [8]. Concern that may arise about the health effects of emitted substances is offset, to some extent, by modern, well-managed WTE plants emitting concentrations of these pollutants lower than the concentrations emitted by MSW traditional incineration [9]. Despite the growing global interest in WTE, the public health implications of RDF combustion remain poorly studied. Currently, there is only one systematic review of the scientific literature on the health impacts associated with atmospheric emissions from WTE processes [10]. The review points out that while the implementation of WTE technologies is increasing, most health studies on incineration do not specifically address the combustion of RDF (different from that of MSW due to the characteristics of the composition of the waste). The review concludes that overall the evidence on the health effects of emissions due to WTE plants is scarce and limited but a rigorous assessment of the technological characteristics of the plants and the type of waste used is necessary as well as monitoring and environmental control both in the design phase and during the plant operations.

As for cement plants, today cement is considered one of the most important building materials in the world and the emissions deriving from the production of this material represent about 5% of global CO_2_ emissions [11]. During the production and transport of cement, gaseous substances and particulates are released into the environment so that workers in cement factories can be exposed to gases and dust deriving from chemical reactions, such as NO_x_, SO_2_, carbon monoxide (CO), CO_2_, dibenzo-p-dioxins polychlorinated and dibenzofurans (PCDD/Fs), polychlorinated biphenyls (PCBs) as well as small amounts of organic compounds, ammonia, chlorine and hydrochloric acid [12–15]. Particularly, people living in the proximity of a cement plant can be exposed to coal, gas, oil, sewage sludge, liquid and solid waste materials and petroleum coke used as energy sources for cement plants [12]. The impact of emissions from a cement plant on neighboring communities also depends on the filtering and abatement systems, the meteorological conditions and the geographical configuration of the area. Exposure to substances emitted by cement factories can induce health effects for both workers and the general population. A recent systematic review [16] has collected results from the last 20 years on the effects of emissions from cement plants on the general population, highlighting risk associations between exposure to cement plants and respiratory symptoms, emphysema, decreased pulmonary function and mortality from respiratory diseases. A study published after the review reported some mortality and hospital admissions excesses, especially among women in the area surrounding a cement plant, compared to neighboring municipalities, for diseases of the circulatory system for which until now the scientific literature reported inadequate evidence [17].

In Italy, the area of the Venafro Valley (Molise region, central-southern Italy) has been characterized since long time by the presence of a WTE and a cement plant. In addition, the valley and the city of Venafro are exposed to heavy and light vehicle traffic, especially due to the presence of two state roads connected with other main city streets in a single intersection in the city centre. Local and regional citizen’s associations committed to environmental and health issues have been rising the alarm over time about the environmental impact, making unsystematic observations of the clusters of diseases and deaths: the first allegations to the Isernia Public Prosecutor’s Office were made in 2009 by the Mothers for Health and the Environment Association. In 2018, a first descriptive study on mortality and hospitalization of residents in the municipalities of Venafro, Pozzilli (where the WTE is located) and Sesto Campano (where the cement plant is located) was promoted by this Association together with the Non-Governmental Organization Doctors for the Environment, ISDE Italy, on data provided by the Molise Region administration. Compared to the regional reference, the study showed mortality and hospitalization excesses for diseases of the circulatory system and excesses only for hospitalizations for respiratory diseases in both sexes (https://www.mammesaluteambiente.it/web/wp-content/uploads/2018/11/Relazione-3-comuni-Molise.pdf?x17222; https://www.mammesaluteambiente.it/; https://www.isde.it/studio-epidemiologico-a-venafro/). The results were presented at a public assembly attended by citizens, associations and local and regional administrators and gave impetus to the realization of an etiological epidemiological study, indicating the retrospective residential cohort design as capable of reconstructing the mortality and morbidity profile in association with the main environmental pressures in the area.

The main goal of the outlined study, called EPIVenafro + 7, was to assess the health risk of residents exposed to pollution produced by the WTE and cement plants, considering risks from both exposure to vehicular traffic and individual risk factors, such as age, sex and socio-economic deprivation. The study, funded by the Basilicata Region through the Venafro Municipality, coordinating seven further Municipalities, is presented in the following pages.

## Methods

The study was carried out in accordance with the Helsinki Declaration of Ethical Principles. All the record linkage procedures between personal, health and environmental data were carried out guaranteeing anonymity, according to precise rules in the management of regional information systems, and in full compliance with current privacy legislation (General Data Protection Regulation - GDPR, European Regulation 2016/679). Personal and health data have been pseudonymized through the same procedure in order to carry out the record linkage procedures and thus obtain the final dataset (minimized for research purposes) containing personal (demographic, residential), exposure and health data. No personal identifiers were sent to the research staff, all addresses were geocoded and the personal data were anonimously analysed.

### Study Design

#### Domain of the study

**Fig. 1:**
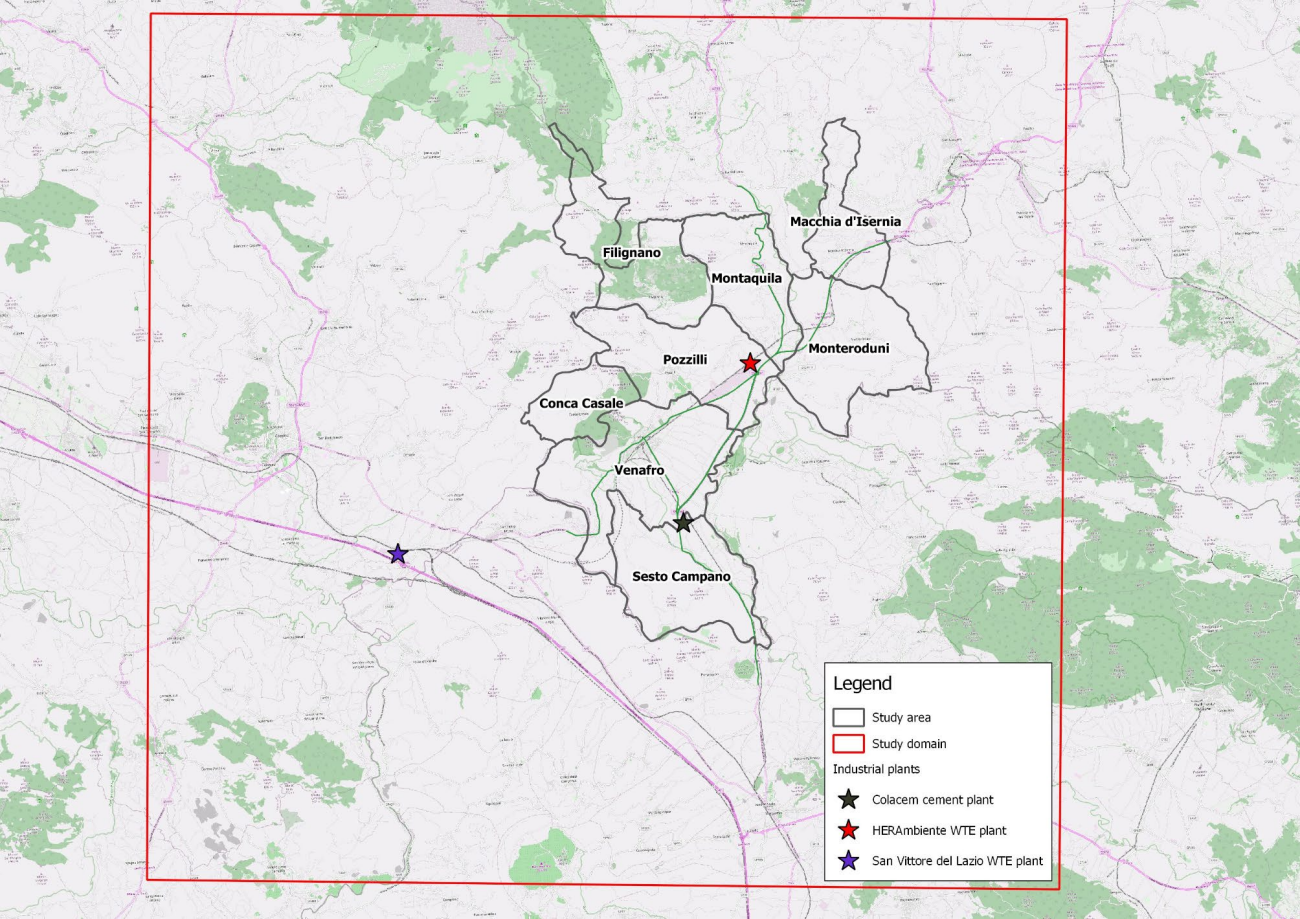
Study domain: the eight municipalities of the Molise Region and the industrial plants considered

Although the first descriptive study was carried out in the three municipalities of Pozzilli, Sesto Campano and Venafro, the domain of the present study included five more municipalities (Conca Casale, Filignano, Montaquila, Monteroduni and Macchia d’Isernia) considered (i) the acquired knowledge, (ii) the orography of the territory, (iii) the location of the plants subject to environmental assessment, (iv) the distribution of roads with greater traffic and (v) the availability of environmental, demographic, socio-economic and health data (Fig. 1).

#### Definition of the cohort and the follow-up period

The retrospective cohort included the total number of inhabitants residing in the eight study municipalities (domain of the study) for at least one year during the follow-up period (2006–2019). Demographic and residential data of all residents were provided by the General Registry Offices of municipalities. The cohort was open and dynamic, i.e. each subject can enter the cohort at different times during the study period considering birth and migration movements. Regarding hospital admissions, we used the methodology also reported in an our previous study [18], i.e. for each hospitalization cause, the calculation of person years (py) concerned the period from the entry in the cohort until the first admission for that cause. In case of change of residence, the total of py was the sum of py accounted for the permanence at each address. Resident subjects died or hospitalized outside the study area but within the study period were considered in the same way as those who died/hospitalized within the study area. For all subjects the residential addresses were georeferenced.

### Exposure assessment

#### The study plants

Three industrial plants were considered (Fig. 1):

- Herambiente WTE plant is located in the municipality of Pozzilli; it is classified both as a Plant Powered by Renewable Sources and as a co-incineration plant and it is active since 2005;
- Colacem S.p.a. is a cement plant active since 2000; it is located in the municipality of Sesto Campano and is intended for the production of building materials and for co-incineration;
- San Vittore WTE plant, active since 2002, is located in the municipality of San Vittore, outside the Venafro Valley. It was included in the study since the population was worried about the potential impact of its plume in the valley.

Plants emit a complex mixture of gaseous and particulate substances. Although different in composition and risk factors for health, they have been conveyed in the same chimneys, generating plumes with strongly correlated atmospheric dispersion. Here, exposure to emissions from industrial plants is estimated using NOx as an indirect measure (proxy) of the average annual spatial distribution of the complex of substances emitted by the plants, also considering that hourly emission measurements of other substances as particulate and SO_2_ showed loss of valid or available values in the reference year.

#### Dispersion modelling system

Reconstruction of air pollution patterns in a valley needs to allow for many physical processes related to the peculiarity of meteorological and dispersive mean flow characteristics of such complex topography. Valleys may be affected by processes such as flow channelling, sheltering, cold-air pooling, drainage, slope flows and plume impingement on higher terrain. Thus, local circulations are superimposed on large scale motions, modifying the mean flow, the turbulence field and dispersion regimes. This requires a careful reconstruction of the 3-D wind, temperature and other meteorological fields in which simulate the transport and dispersion of pollutants in a typical weather year representative for a longer period. Due to the terrain complexity we use a modelling chain which merges the prognostic meteorological model WRF (Weather Research and Forecasting model) to provide large scale mean flows, the micrometeorological model CALMET [19] and the dispersion model CALPUFF [20, 21]. Horizontal domains and grid sizes were designed considering both computational time limitations and the capability of the model to resolve essential mesoscale and locale features over the area. WRF meteorological data for synoptic-scale meteorology for the reference year 2016 were provided on a domain centred on the municipality of Venafro of 100 km*100 km large enough to reproduce large-scale circulation, with a grid size of 1 km. Reasons for selection of 2016 as a reference year were two: the availability of hourly plant emissions data and the absence of weather anomalies for the year. Combustion-generated plant’s hot plumes contain mixture of gaseous and particulate substances, not all monitored. Analysis of the available emissions data yielded the selection of NO_x_ as a surrogate measure of contaminants emitted by the industrial plants. As input to the dispersion model, hourly NO_x_ emissions for the Pozzilli and Sesto Campano plants provided by the operators were used, while for the San Vittore plant, the authorized data were considered (Table 1).

**Table 1:** Yearly averaged emission of nitrogen oxides for the three plants

Plant	Stack height h (m)	Yearly averaged exit speed V (m/s)	Flue-gas temp. T (K)	Nitrogen oxides NO_x_ (g/s)
Herambiente	43	19	427	3.9
Colacem	106	9	391	25
San Vittore (stack 2,3)	50,50	20,19	406,404	0.6,0.5

Micrometeorological data and annual average NO_x_ concentration were then estimated on a smaller domain of 45 km*43 km with a grid size of 500 m*500m (Table 2).

**Table 2:** CALMET model setup

	CALMET
Horizontal domain	45 km x 43 km
Grid size	0.5 km x 0.5 km
Number grid points Nx, Ny	120, 120
Number of vertical levels	10
First vertical level	20 m
Vertical domain	4,000 m

**Fig. 2:**
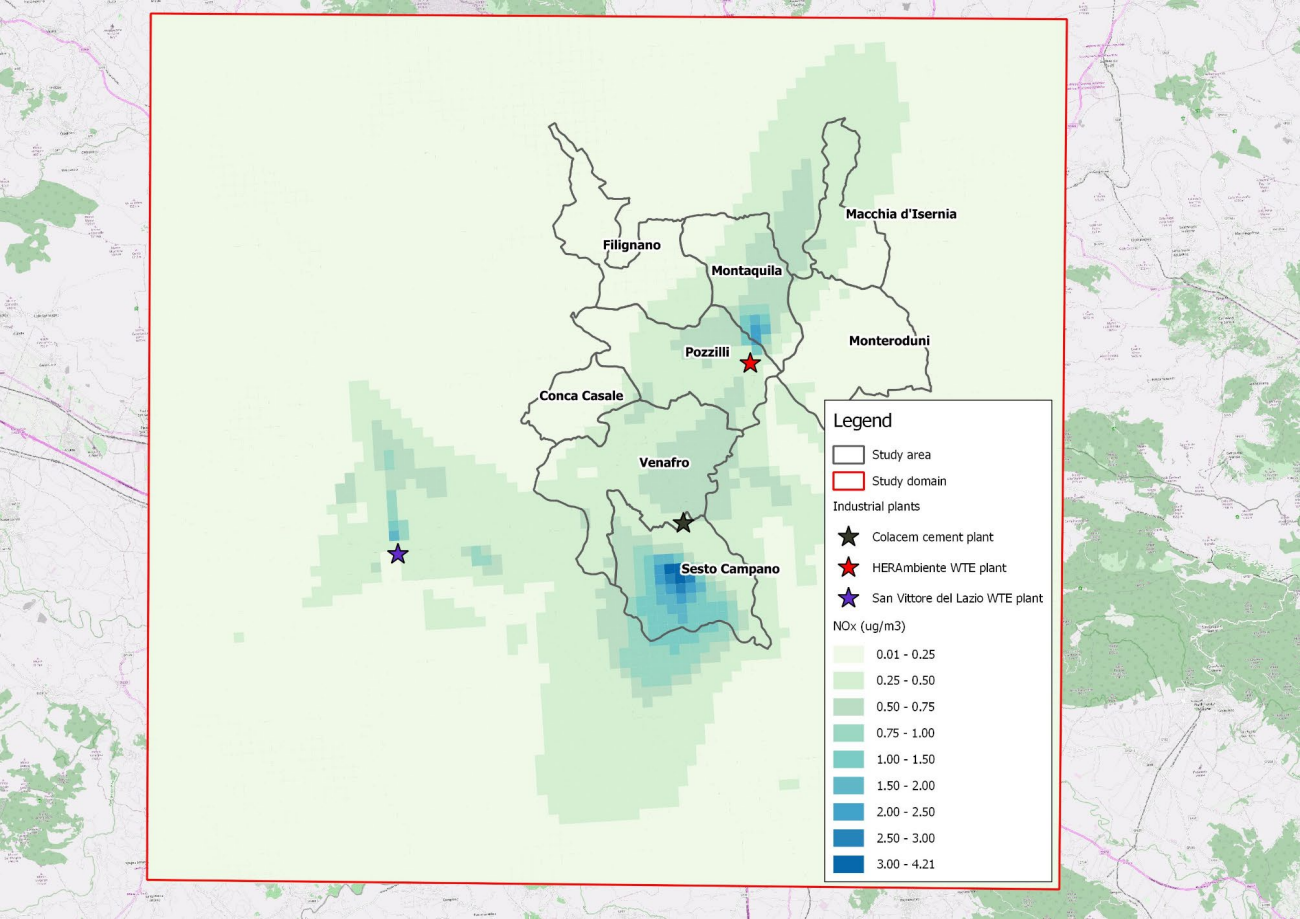
Dispersion map on the territory of nitrogen oxides (NOx) with the WRF/CALMET/CALPUFF model (reference year 2016)

Figure 2 shows the spatial average of ground-level of NO_x_ concentrations with the highest values occurring in the area close to the plants, particularly on the slopes where the emitted plumes appear to impinge. On annual average, the contribution of the San Vittore incinerator on the valley appears negligible. The distribution is consistent with the prevailing wind directions and the complexity of the area. The average concentration is of 0.19 µg/m^3^ (standard deviation of 1.23 µg/m^3^) with a minimum and a maximum of 0.01 µg/m^3^ and 4.2 µg/m^3^, respectively. The dispersion map can be considered a significant footprint of industrial emissions by which to determine an effective classification between the less and the more exposed.

#### Population exposure

For each subject in the cohort, the estimated NO_x_ concentration at the residence address was assigned to each georeferenced address. Each individual exposure was then classified according to four levels of NO_x_ exposure from plants using the “*natural break*” method which is the most appropriate method given the population exposure distribution and the plants location. This method groups the individual exposure data into classes, trying to minimize the average deviation of each class from the average of the class, maximizing the deviation of each class from the average of the other classes; in more technical terms, the method identifies the levels that minimize the variance within classes and maximize the variance between classes [22].

**Fig. 3:**
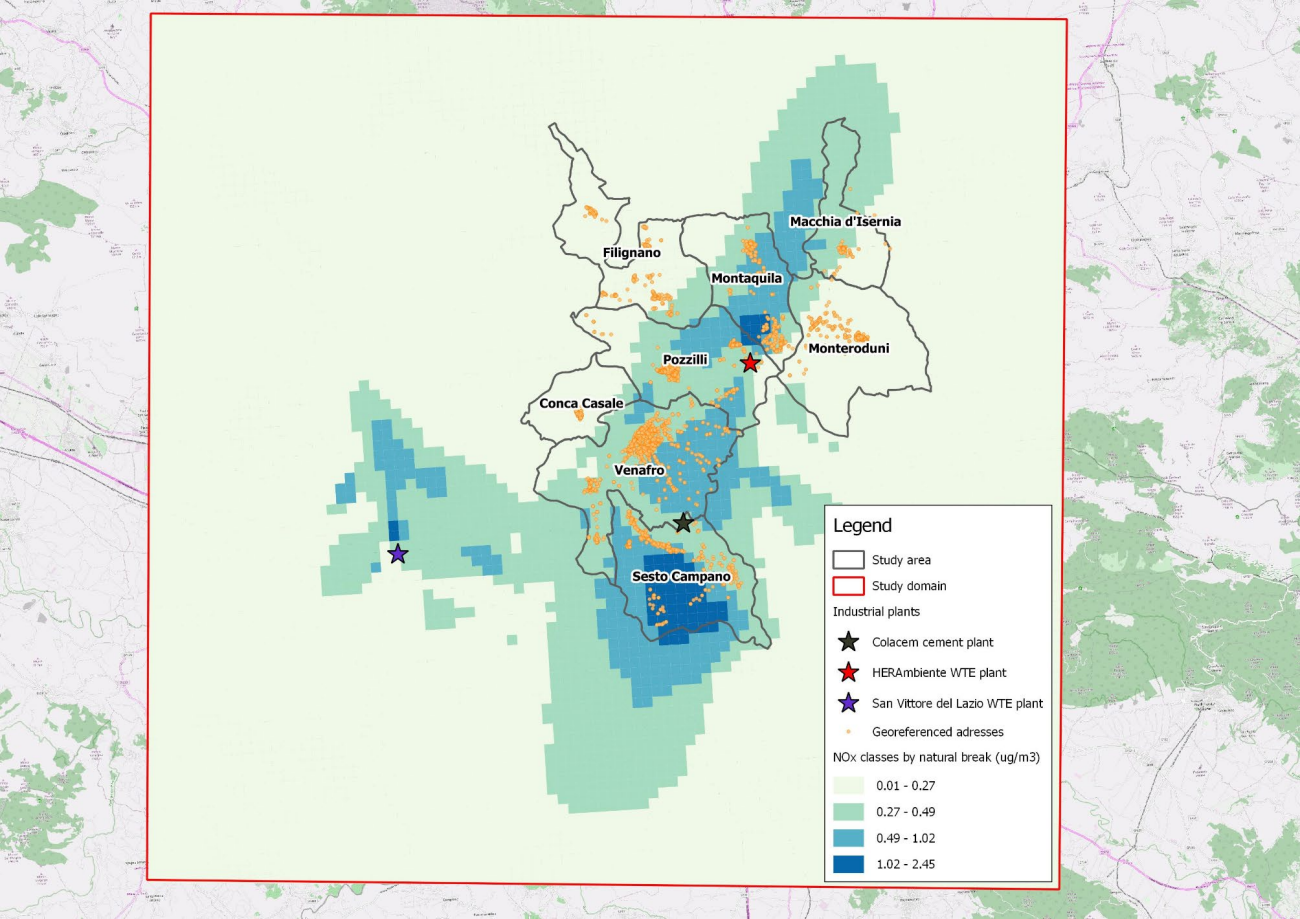
Exposure class to the cohort through the dispersion map (four classes defined for nitrogen oxides - NOx)

In particular, the four exposure classes were defined according to the following levels (Fig. 3):

- class 1 (less exposed; class of reference): 0.01–0.27 µg/m^3^;
- class 2: 0.27–0.49 µg/m^3^;
- class 3: 0.49–1.02 µg/m^3^;
- class 4 (class with higher exposure): 1.02–2.45 µg/m^3^.

#### Exposure to intensive vehicular traffic

**Fig. 4:**
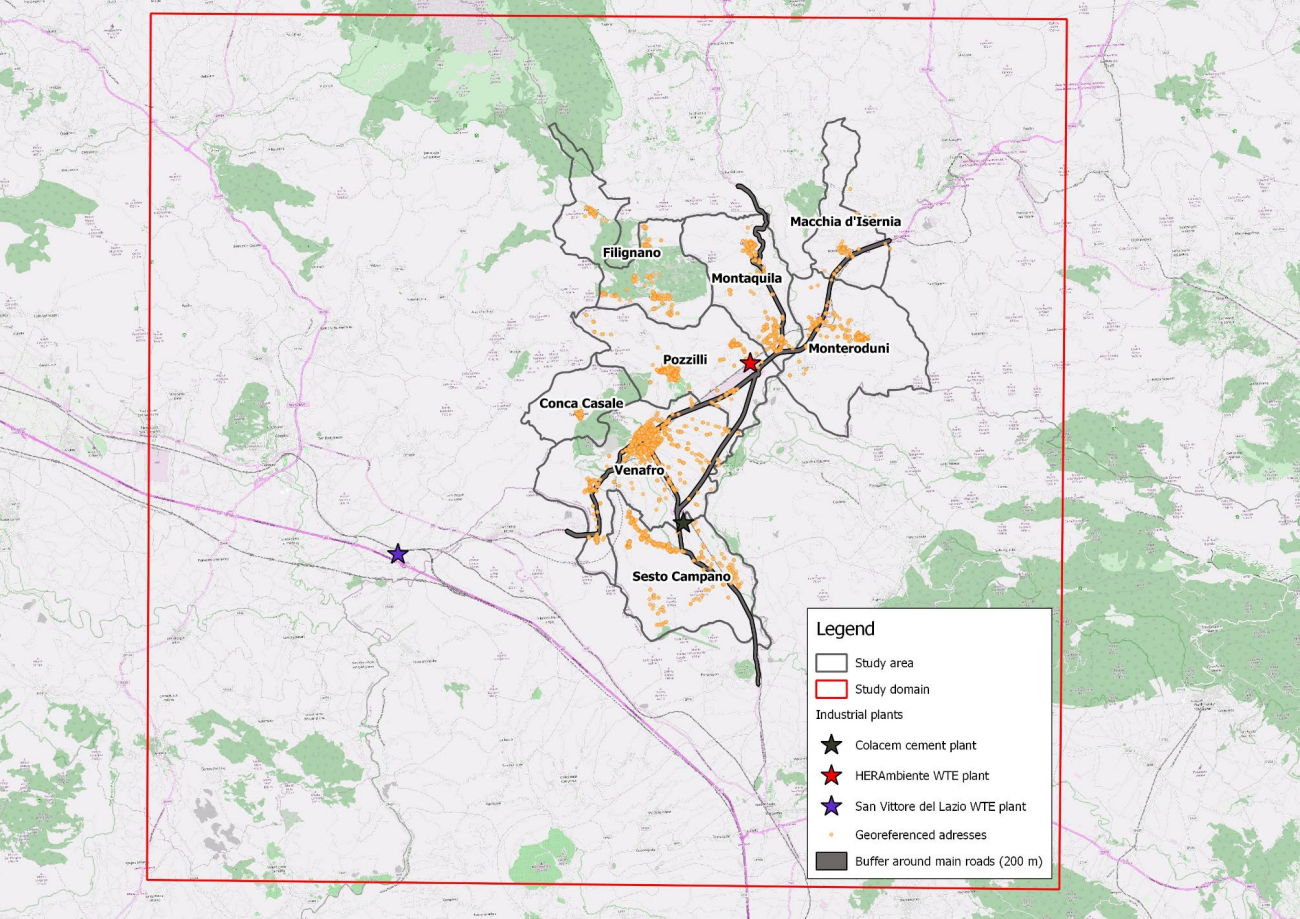
Exposure to vehicular traffic on the main roads with a buffer of 100 m

In order to define the exposure of the study population to vehicular traffic, the distance to linear sources was used as a proxy for environmental exposure. In fact, in case of lack of availability of diffusion maps, the distance from the sources can be considered as a proxy of environmental exposure. This method was used to define an exposure of the study population to vehicular traffic which exerts significant environmental pressure on the entire area under study and in particular on the city of Venafro. A buffer of 200 m (100 m to the right and left of the road centre line) has been calculated for the main state roads that insist on the territory. The presence of the cohort subjects within the buffer area was used as exposure to vehicular traffic on the main roads in the area (Fig. 4).

#### Exposure to socio-economic factors

Socio-economic status was considered as a confounder of the relationship between exposure to air pollutants and the health outcomes considered, as it is well recognized as being associated with both factors. The socio-economic Deprivation Index (DI) calculated at the census section level using the population census was considered as a proxy of socio-economic factors. DI was calculated for each census section on the basis of five variables collected at the 2011 population census by the National Statistical Institute [23]:

- percentage of the population with an education equal to or lower than the elementary school certificate (failure to achieve the compulsory schooling);
- percentage of the active population unemployed or seeking their first job;
- percentage of occupied rented dwellings;
- percentage of single-parent families with cohabiting dependent children;
- population density (number of occupants per 100 m^2^).

**Fig. 5:**
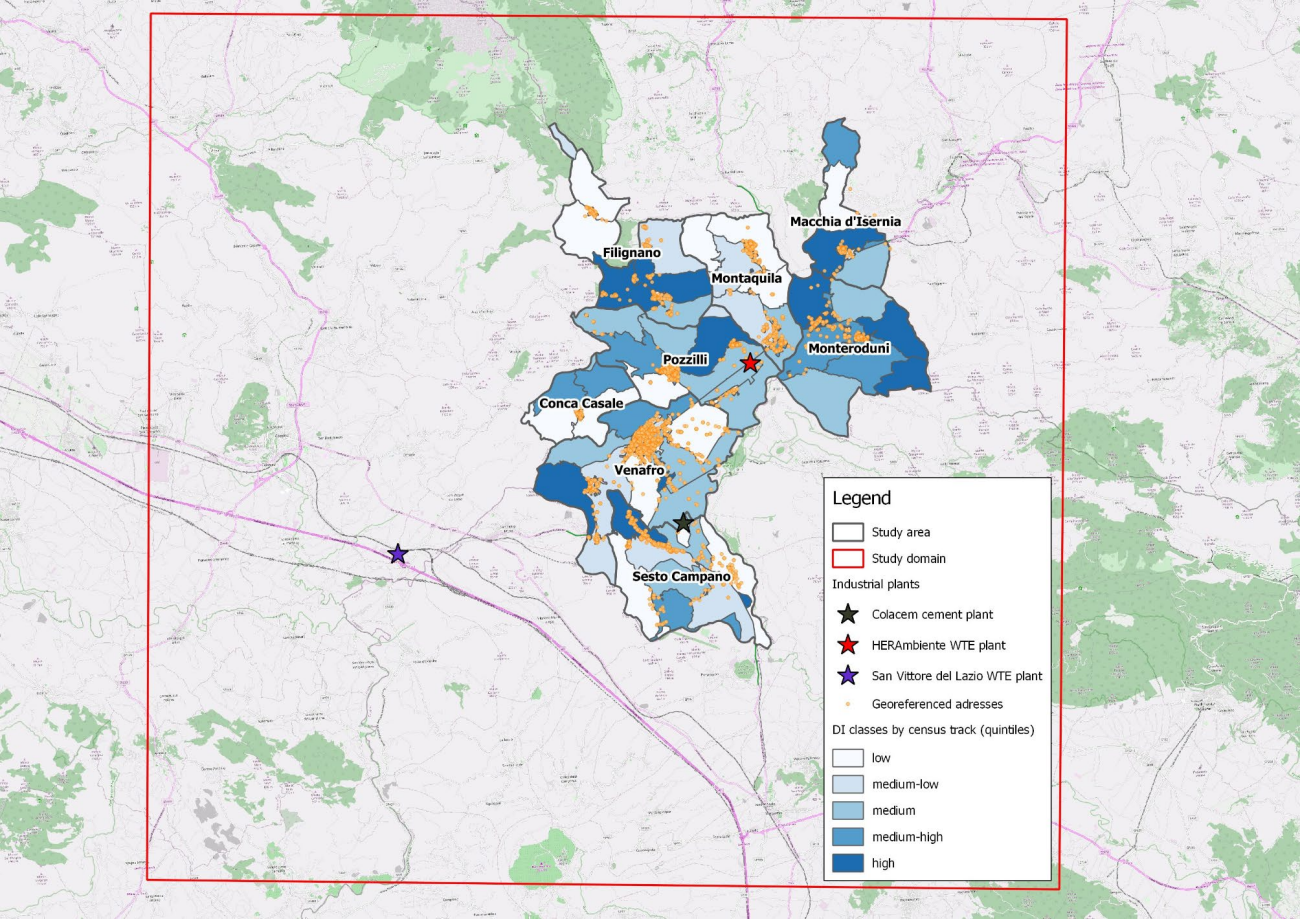
Categorical socio-economic deprivation index (DI) distribution in relation to the area of the eight study municipalities

The DI is a continuous variable representing the distribution of the differences in socioeconomic deprivation of each census section of the study area compared to the regional average deprivation. Each subject was assigned the DI value of the census section in which their first residence fell. The distribution of the cohort DIs was divided into five deprivation classes (high, medium-high, medium, medium-low, low) according to the levels defined by the quintiles of the distribution (Fig. 5).

### Health outcomes

The following health data were provided in pseudonymised form by the Molise Regional Health Agency:

- mortality data for the period 2006–2019. Source: Nominative Cause of Death Register. Data were coded using the International Classification of Diseases 10th revision (ICD-10);
- hospitalization data for the period 2006–2019. Source: Hospital Discharge Forms (HDFs) of the Molise Region (hospitalizations of residents occurring inside and outside the region). In order to estimate only new hospitalizations (incident cases), for each cause of hospitalization under study, the first admission occurred during the follow-up period was selected using data from the regional hospital information system (including extra-regional mobility). Data were coded using the International Classification of Diseases 9th revision (ICD-9).

The subjects included in the cohort were associated, through a pseudonymised code, with the first admission for each cause of hospitalization under study, as well as for the death (if any). The age at diagnosis at discharge was calculated by the difference between date of admission and date of birth.

On the basis of the knowledge on the health risks potentially associated with air pollution from WTE and cement plants, diseases of the circulatory and respiratory system were identified as the main disease groups to be studied. The short to medium induction-latency period compatible with the 2006–2019 study period also played a role in this choice.

Specifically, the causes selected were:

- Diseases of the circulatory system (ICD-9 Codes 390–459; ICD-10 Codes I00-I99);
  - Heart diseases (ICD-9 Codes 390–429; ICD-10 Codes I00-I52);
  - Ischemic Heart Diseases (IHD) (ICD-9 Codes 410–414; ICD-10 Code I20-I25);
    - Acute Myocardial Infarction (AMI) (ICD-9 Code 410; ICD-10 Code I21);
  - Cerebrovascular diseases (ICD-9 Codes 430–438; ICD-10 Codes I60-I69);
- Diseases of the respiratory system (ICD-9 Codes 460–519; ICD-10 Codes J00-J99);
  - Acute respiratory diseases (ICD-9 Codes 460–466, 480–487; ICD-10 Codes J00-J06, J10-J18, J20-J22);
  - Chronic diseases of the lower respiratory tract (except asthma) (ICD-9 Codes 490–492, 494, 496; ICD-10 Codes J40-J44, J47);
  - Asthma (ICD-9 Code 493), considered only in the morbidity analyses.

### Statistical analysis

A multiple time-dependent and sex-specific Cox regression model was used to study risk association between exposures and specific causes of mortality/morbidity. By this model, the risk associations were calculated within the study area as Hazard Ratio (HR) between the risk of death/hospitalization of the population in the most exposed classes and the risk of death/hospitalization of the population in the class with less exposure. The HRs were adjusted by age groups (0–44; 45–54; 55–64; 65–74; 75–84, 85+), proximity or not to the roads selected (dichotomous factor) and the five classes of DI previously defined. The HRs were accompanied, both by the 95% Confidence Interval (95%CI) and by the strength of empirical evidence in favor of the risk association hypothesis (1-*p*, with values between 0 and 1). The 1-*p* indicator represents a probabilistic measure of credibility of the estimated risk association hypothesis: the higher the 1-*p*, the greater the strength of the evidence towards a hypothesis of health risk associated with the exposure under study [24]. For each cause of death and hospitalization under study, the trend of mortality/morbidity risks were also calculated as the NO_x_ increased by 1 µg/m^3^, accompanied by the 1-*p* value. Schoenfeld’s test to evaluate the proportional hazard assumption in all Cox regression models was also carried out. Analyses were performed separately by sex using STATA v.15 [StataCorp. 2017. Stata Statistical Software: Release 15. College Station, TX: StataCorp LLC].

## Results

Table 3 shows descriptive cohort characteristics (sex, age, socio-economic status and proximity to the roads selected) from mortality data. Tables 4 and 5 show the relationship between model-estimated NO_x_ exposure and mortality/morbidity causes, considering four NO_x_ exposure classes (from the highest exposure, class 4, to the less exposed, class 1, as reference) for men and women, separately. The risk trend for increments of 1 µg/m^3^ of NO_x_ is also reported. For all the mortality and hospitalization causes, results of Schoenfeld’s test allowed accepting the hypothesis of proportional hazards (for all test *p* > 0.05).

**Table 3:** Descriptive cohort characteristics by risk factors and mortality data (period 2006–2019)

Cohort		Circulatory system				Respiratory system			
		PY	Deaths (CS)	CR x 1000 PY	95%CI	PY	Deaths (RS)	CR x 1000 PY	95%CI
	*Total*	317,810	1,302			317,841	215		
**Sex**	*Men*	156,030	576	3.69	3.40–4.01	156,056	130	0.83	0.70–0.99
	*Women*	161,780	726	4.49	4.17–4.83	161,785	85	0.53	0.42–0.65
**Age classes (years)**	*0–44*	138,462	15	0.11	0.07–0.18	138,474	4	0.03	0.01–0.08
	*45–54*	45,893	28	0.61	0.42–0.88	45,893	0		
	*55–64*	45,222	51	1.13	0.86–1.48	45,234	8	0.18	0.09–0.35
	*65–74*	38,579	130	3.37	2.84–4.11	38,578	25	0.65	0.44–0.96
	*75–84*	29,899	441	14.75	13.55–16.32	29,903	87	2.91	2.36–3.59
	*85+*	19,755	637	32.25	30.43–35.68	19,759	91	4.61	3.75–5.66
**Classes of socio-economic deprivation (DI)**	*Low*	80,249	280	3.48	3.10–3.91	80,434	46	0.57	0.43–0.76
	*Medium-low*	111,424	447	4.01	3.66–4.40	111,438	77	0.69	0.55–0.86
	*Medium*	34,939	162	4.64	3.97–5.41	34,939	21	0.60	0.39–0.92
	*Medium-high*	53,623	234	4.36	3.84–4.96	53,624	38	0.71	0.52–0.97
	*High*	37.395	179	4.79	4.13–5.54	37,406	33	0.89	0.63–1.25
**Classes of exposure to nitrogen oxides (NO**_**x**_) **(µg/m**^**3**^**)**	*Class 1 (ref.): 0.01–0.27*	55,700	264	4.47	4.20–5.35	55,701	54	0.97	0.74–1.27
	*Class 2: 0.27–0.49*	207,487	741	3.57	3.32–3.84	207,515	124	0.60	0.50–0.71
	*Class 3: 0.49–1.02*	51,279	278	5.42	4.82–6.10	48,351	33	0.68	0.49–0.95
	*Class 4: 1.02–2.45*	3,344	19	5.68	3.62–8.91	6,274	4	0.64	0.15–2.39
**Proximity to selected roads**	*Yes*	69,809	244	3.50	3.08–3.96	69,821	39	0.56	0.41–0.76
	*No*	248,004	1,058	4.27	4.02–4.53	248,020	176	0.71	0.61–0.82

Notes – PY: Person-Years; CS: Circulatory System; CR: Crude Rate; 95%CI: Confidence Interval at 95% of probability; RS: Respiratory System

**Table 4:** Mortality risk association analysis (period 2006–2019) by sex and class of nitrogen oxides (NO_x_) exposure

Cause (ICD-10 code)	Exposure class	MEN				WOMEN			
		n	HR	*1-p*	CI95%	N	HR	*1-p*	CI95%
**Diseases of the circulatory system** **(I00-I99)**	1 (ref.)	113				151			
	2	334	1.18	0.835	0.94–1.48	407	1.07	0.483	0.87–1.31
	3	118	1.37	0.977	1.04–1.79	160	1.27	0.956	1.01–1.60
	4	11	1.35	0.654	0.72–2.54	8	0.68	0.700	0.33–1.40
	Trend		1.40	0.919	0.96–2.05		0.96	0.187	0.66–1.38
**Heart diseases** **(I00-I52)**	1 (ref.)	68				79			
	2	225	1.28	0.905	0.96–1.72	265	1.33	0.963	1.02–1.75
	3	71	1.32	0.883	0.93–1.87	101	1.49	0.989	1.10–2.04
	4	10	1.95	0.946	0.99–3.85	6	0.94	0.114	0.41–2.18
	Trend		1.51	0.922	0.95–2.39		1.18	0.554	0.77–1.83
*Ischemic Heart Diseases (IHD)* *(I20-I25)*	1 (ref.)	24				23			
	2	66	0.99	0.032	0.60–1.64	66	1.10	0.286	0.66–1.84
	3	18	0.90	0.256	0.47–1.71	25	1.01	0.035	0.56–1.85
	4	3	1.69	0.593	0.49–5.79	< 3			
	Trend		1.40	0.542	0.57–3.41		n.c.		
*Acute Myocardial Infarction (AMI)* *(I21)*	1 (ref.)	10				10			
	2	22	0.73	0.542	0.32–1.67	23	0.81	0.393	0.36–1.81
	3	6	0.52	0.774	0.18–1.51	6	0.56	0.714	0.19–1.63
	4	< 3				< 3			
	Trend		n.c.				n.c.		
*Cerebrovascular diseases* *(I60-I69)*	1 (ref.)	21				42			
	2	75	1.60	0.924	0.95–2.68	113	1.08	0.293	0.74–1.57
	3	38	2.50	0.999	1.44–4.35	49	1.41	0.885	0.92–2.17
	4	< 3				< 3			
	Trend		n.c.				n.c.		
**Disease of the respiratory system** **(J00-J99)**	1 (ref.)	32				22			
	2	78	0.93	0.238	0.60–1.46	46	0.90	0.304	0.52–1.55
	3	19	0.74	0.674	0.41–1.34	16	1.01	0.033	0.52–1.99
	4	< 3				< 3			
	Trend		n.c.				n.c.		
*Acute respiratory diseases* *(J00-J06; J10-J18; J20-J22)*	1 (ref.)	10				4			
	2	27	1.11	0.205	0.50–2.47	14	1.34	0.380	0.42–4.26
	3	5	0.73	0.415	0.24–2.23	8	2.31	0.815	0.67-8.00
	4	< 3				< 3			
	Trend		n.c.				n.c.		
*Chronic diseases of the lower respiratory tract (except asthma)* *(J40-J44; J47)*	1 (ref.)	9				11			
	2	27	1.19	0.331	0.53–2.69	12	0.51	0.843	0.20–1.30
	3	7	0.92	0.134	0.33–2.55	< 3			
	4	< 3				< 3			
	Trend		n.c.				n.c.		

Notes –The analyses were performed in the period 2006–2019 and adjusted for age classes, proximity or not to the selected roads and socio-economic deprivation index. ICD10: International Classification of Diseases, 10th revision; n: numerosity; HR: Hazard Ratio; CI95%: Confidence Interval at 95% of probability; 1-*p*: strength of evidence in favour of an excess/defect of mortality risk; Trend: risk trend for 1 µg/m^3^ NO_x_ increment; 1 (ref.): exposure class 1, reference (0.01–0.27 µg/m^3^); 2: exposure class 2 (0.27–0.49 µg/m^3^); exposure class 3 (0.49–1.02 µg/m^3^); exposure class 4 (1.02–2.47 µg/m^3^); n.c.: not calculated

**Table 5:** Hospitalization risk association analysis (period 2006–2019) by sex and class of nitrogen oxides (NO_x_) exposure

Cause (ICD-9 code)	Exposure class	MEN				WOMEN			
		n	HR	*1-p*	CI95%	n	HR	*1-p*	CI95%
**Diseases of the circulatory system** **(390–459)**	1 (ref.)	418				369			
	2	15	1.15	0.984	1.03–1.30	1,339	1.23	0.999	1.09–1.39
	3	409	1.12	0.871	0.97–1.29	343	1.14	0.900	0.98–1.33
	4	26	0.87	0.498	0.58–1.30	21	0.93	0.266	0.59–1.44
	Trend		1.07	0.457	0.86–1.33		0.96	0.291	0.75–1.21
*Heart diseases* *(390–429)*	1 (ref.)	280				233			
	2	965	1.02	0.249	0.89–1.18	795	1.10	0.751	0.94–1.28
	3	262	1.03	0.281	0.86–1.23	204	1.04	0.302	0.85–1.27
	4	19	0.99	0.018	0.62–1.59	17	1.26	0.632	0.76–2.07
	Trend		1.01	0.044	0.76–1.34		0.97	0.125	0.71–1.34
*Ischemic Heart Diseases (IHD)* *(410–414)*	1 (ref.)	121				76			
	2	466	1.09	0.582	0.88–1.35	248	0.97	0.147	0.74–1.29
	3	139	1.24	0.901	0.96–1.61	69	0.95	0.245	0.67–1.34
	4	9	1.12	0.249	0.56–2.22	10	2.04	0.961	1.04–4.02
	Trend		1.19	0.611	0.80–1.76		1.42	0.835	0.87–2.34
*Acute Myocardial Infarction (AMI)* *(410)*	1 (ref.)	51				32			
	2	194	1.12	0.499	0.81–1.56	81	0.76	0.776	0.49–1.18
	3	65	1.46	0.946	0.99–2.16	29	0.88	0.349	0.52–1.51
	4	< 3				6	2.48	0.951	1.00-6.12
	Trend	nc					1.70	0.848	0.82–3.53
*Cerebrovascular diseases* *(430–438)*	1 (ref.)	147				147			
	2	569	1.18	0.909	0.97–1.44	569	1.27	0.984	1.04–1.54
	3	158	1.19	0.849	0.94–1.50	145	1.16	0.779	0.91–1.48
	4	8	0.78	0.493	0.38–1.61	9	0.98	0.049	0.50–1.93
	Trend		1.07	0.284	0.74–1.55		0.89	0.454	0.61–1.3
**Diseases of the respiratory system** **(460–519)**	1 (ref.)	196				176			
	2	815	1.18	0.948	1.00-1.39	623	0.99	0.111	0.82–1.18
	3	192	1.07	0.456	0.87–1.32	152	0.98	0.117	0.78–1.24
	4	19	1.43	0.854	0.88–2.31	6	0.63	0.728	0.28–1.43
	Trend		1.13	0.555	0.83–1.53		0.80	0.728	0.53–1.19
*Acute respiratory diseases* *(460–466; 480–487)*	1 (ref.)	76				69			
	2	282	1.08	0.423	0.82–1.42	188	0.73	0.960	0.54–0.99
	3	71	1.06	0.269	0.75–1.49	50	0.81	0.702	0.55–1.20
	4	8	1.59	0.779	0.76–3.35	< 3			
	Trend		1.15	0.422	0.70–1.91		0.46	0.948	0.21–1.01
*Chronic diseases of the lower respiratory tract (except asthma)* *(490–492; 494; 496)*	1 (ref.)	31				26			
	2	131	1.34	0.830	0.88–2.03	75	0.91	0.306	0.56–1.47
	3	31	1.11	0.298	0.66–1.86	25	1.21	0.487	0.68–2.15
	4	5	2.27	0.903	0.86-6.00	3	2.37	0.834	0.70–8.04
	Trend		1.43	0.688	0.71–2.86		1.58	0.72	0.69–3.64
*Asthma* *(495)*	1 (ref.)	6				5			
	2	14	0.55	0.750	0.20–1.52	17	0.68	0.529	0.23–1.96
	3	8	1.09	0.113	0.34–3.52	4	0.82	0.228	0.21–3.22
	4	< 3				< 3			
	Trend	nc				nc			

Notes – The analyses were performed in the period 2006–2019 and adjusted for age classes, proximity or not to the selected roads and socio-economic deprivation index. ICD9: International Classification of Diseases, 9th revision; n: numerosity; HR: Hazard Ratio; CI95%: Confidence Interval at 95% of probability; 1-*p*: strength of evidence in favour of an excess/defect of hospitalization risk; Trend: risk trend for 1 µg/m^3^ NO_x_ increment; 1 (ref.): exposure class 1, reference (0.01–0.27 µg/m^3^); 2: exposure class 2 (0.27–0.49 µg/m^3^); exposure class 3 (0.49–1.02 µg/m^3^); exposure class 4 (1.02–2.47 µg/m^3^); n.c.: not calculated

Some indications on the results interpretations are provided below:

- HRs based on less than 3 cases were not reported both for privacy reasons and due to the high inaccuracy of the estimates;
- to highlight the results for the most NO_x_ exposed-classes, only those significant results referring to classes 3 and 4 of exposure were commented;
- comments on any risk excesses observed in the most exposed classes are reported always referencing to the exposure class at the lowest level, i.e. the reference class (class 1);
- the results were commented with the support of the observed probability value (1-*p*, as previously defined), in accordance with the recent scientific literature which recommends going beyond the sole concept of statistical significance, also reporting the risk associations which, although not statistically significant, may be indicative of critical issues to be further investigated by reducing uncertainty [25].

### Descriptive analysis

During the period 2006–2019, the cohort counted 29,495 subjects and 317,810 py, of which 14,804 men (50.2%), with an average follow-up time of 10.7 years (10.5 and 11.0 years for men and women, respectively). For cardiovascular diseases, there were 1,302 deceased subjects of which 576 men (44.2%); for respiratory diseases the number of deceased subjects was 215 of which 130 men (60.5%).

The mortality rate for the diseases of the circulatory system was higher among women and significantly increased with increasing age classes and DI (Table 3). The mortality rate of the classes with higher exposure is higher than the reference class of NO_x_ exposure (Table 3). Subjects not living in the proximity of the roads selected showed a higher mortality rate for diseases of the circulatory system (Table 3); subjects in the reference class of NO_x_ exposure and those living not in proximity of the selected roads, showed the higher mortality rate for the diseases of the respiratory system (Table 3).

The mortality rate for respiratory diseases was higher among men and increased with increasing age classes but not with DI increasing (Table 3).

### Results of mortality analysis

Excess mortality from diseases of the circulatory system, for men (+ 37%; 97.7% of evidence probability in favour of the hypothesis of risk association (1-*p*)) and for women (+ 27%; 1-*p* 95.6%) were observed in class 3. In the highest exposure class, based on a small number of cases, there was a risk excess among men and a risk defect among women. Among men a + 40% increased risk trend (1-*p* 92%) was observed for 1 µg/m^3^ NO_x_ increments (Table 4).

For heart diseases, class 3 risk excesses were also observed in both sexes (men: +32%; 1-*p* 88.3%; women: +49%; 1-*p* 98.9%). In the highest exposure class an excess risk of + 95% (1-*p* 94.6%) was observed among men (estimation based on a small number of cases); among women no excess risk was observed. Furthermore, among men a + 51% increasing risk trend, for increments of 1 µg/m^3^ NO_x_ (1-*p* 92.2%) was observed (Table 4).

For cerebrovascular diseases, in class 3 the excess risk observed among men was higher than that observed among women (men: +150%; 1-*p* 99.9%; women: +41%; 1-*p* 88.5%) (Table 4).

No risk excesses/defects were observed for any disease of the respiratory system, except a risk excess for acute respiratory diseases among women in class 3 based on a small number of cases (+ 131%; 1-*p* 81.5%) (Table 4).

### Results of hospital admission analysis

Considering diseases of the circulatory system, risk excesses of + 12% (87.1% of evidence probability in favour of the hypothesis of risk association (1-*p*)) and + 14% (1-*p* 90.0%) were observed in class 3 among both men and women, respectively. No noteworthy signals were observed in the highest exposure class (Table 5). Risk excesses were observed in all sub causes of the circulatory system, except for heart diseases. Specifically, for IHD and AMI, among men in class 3, risk excesses of + 24% (1-*p* 90.1%) and + 46% (1-*p* 94.6%) were observed, respectively. For the same aforementioned sub-causes, among women in class 4, risk excesses based on a small number of cases were observed, specifically + 104% (1-*p* 96.1%) for the IHD and + 148% (1-*p* 95.1%) for AMI (Table 5). For cerebrovascular disease, for both sexes, risk excesses in class 3 were observed (men: +19%, 1-*p* 84.9%; women: +16%, 1-*p* 77.9%) (Table 5).

## Discussion

The aim of the EPIVenafro + 7 study was to assess the health risk of residents of eight municipalities of Venafro Valley (Molise region) exposed to different sources of industrial pollution present in the area (WTE plants and a cement plant) considering individual risk factors, such as age, sex, proximity to specific roads and socio-economic deprivation.

The adopted design does not allow to exclude that in the investigated area, in addition to the effects of the pollution sources considered, there may be an effect from other environmental pressures that could influence the relationship under study. It should be noted that the map of the NO_x_ proxy modelled for the study provides indications of a not significant contribution from the San Vittore WTE plant.

Mortality analyses highlighted risk excess for diseases of the circulatory system in both sexes that seem to be attributable, in particular, to heart and cerebrovascular diseases. As regards the hospitalization analyses, excesses were observed for diseases of the circulatory system in both sexes, in particular for ischemic and cerebrovascular diseases and AMI and for respiratory diseases only among the most exposed men with some signals for acute and chronic respiratory diseases among both sexes in the highest exposed class.

It should be outlined that for both mortality and hospitalization, the most significant excesses, mentioned above, emerged for the medium-high exposure class and not for the highest class, a result that should be duly considered. This result could be due to the methodological choice of using natural breaks which resulted in a low number of py in class 4 and consequently a low power in highlighting significant excesses in that class.

The scientific literature considering the general population health effects of exposure to emissions from WTE/cement production plants is currently scarce, produces limited evidence and studies are characterized by some limitations that may lead to bias.

The direct measurement of the health effects of WTE plants emissions due to the combustion of RDF has been little studied, partly due to the difficulty in quantifying the effects on the health of populations deriving from low or generally not accurately estimated exposure levels [26]. To date, only a systematic review [10] analysed the health impacts deriving only from emissions from WTE plants. In this review there were only two epidemiological studies, which however studied different outcomes from those of EpiVenafro + 7 study, such as the presence of metals in the urine [27] or focus on sub-groups of populations (children) [28]. The review also included health impact/risk assessment studies according to which, under normal operating conditions, limited evidence was observed for an increase in carcinogenic and non-carcinogenic effects, given that WTE plants are usually characterized by very low emissions compared to classic incinerators [29–31]. Other reviews analysed the effects on the health of the general population and workers deriving from exposures to incinerator of first generation (plants active until 1989 - first European directive on waste incineration, 89/429/EEC), second generation (plants active between 1989 and 2006 – period of transition) and third generation (plants active after 2006 - publication of BAT REF Waste incineration) [26, 32, 33]. All these studies agree that the available evidence on the health effects investigated in the general population living near incinerators showed no consistent excess risk. Data on plants of both first and second generation showed that, if there were any excesses at all, these were at most modest and that direct evidence from third generation plants is scarce and only related to selected short-term outcomes [26]. On the one hand, the methodological limitations of the available data do not allow to firmly conclude for an absence of any health effect of modern incinerators and on the other hand, no strong and consistent signal emerged from the available literature [26, 33]. There is a need to overcome the design weaknesses of previous studies, such as the study design (i.e. ecological study potentially leads to the “ecological fallacy”) and the exposure assessment which is usually evaluated at the place of residence, thus ignoring variations arising from individuals who spent a substantial portion of time away from the residence [26]. Furthermore, all studies ignored characteristics of the residence and lifestyle habits that can affect exposure; thus, information on some potentially important confounding factors (e.g., smoking and other lifestyle habits, and individual level social class) is lacking in most studies. Negri and colleagues (2020) concluded that additional monitoring of health effects should be carried out tending to provide both more precise measures of exposure, including the use of dispersion models and geocoding of addresses, and newer health databases in order to incorporate more information on potential confounding factors. In addition, also the health impact assessments based on quantitative estimates of pollutants may provide further information [33].

The systematic review by Raffetti et al. (2019) on the effects of cement plants emissions on the health of the general population counted 17 epidemiological studies, 5 of these based exclusively on children, 4 based exclusively on questionnaires, 4 assessed outcomes different from those of EpiVenafro + 7 study [16]. Among the other four studies, 3 based the exposure assessment on the distance from the plant [34, 35] or on the residence in the city where the plant is located [36]. Only one study, conducted in the Po Valley, used the same pollutant proxy as our study, i.e. NO_x_, reporting an association between cement plant exposure and the risk of hospitalization for respiratory and cardiovascular diseases [37]. In this study, authors used an exposure map obtained with a very different methodology from our, essentially based on measurements in the environment (multi-source) through a winter measurement campaign of only 20 days in four measurement points. The result was three exposure areas with reference values from about 80 µg/m^3^ to about 200 µg/m^3^ [37]. Despite this profound difference in the exposure definition, our results were partially in line with those of Bertoldi et al. (2012) although we found a hospitalization risk excess only for cardiovascular diseases. Another study using the distance from the plant as a proxy for the exposure assessment reported results in line with ours regarding mortality from cerebrovascular diseases among men [36]. The same study observed excess mortality risk from respiratory diseases [36] while we found excess hospitalization risk. After the review by Raffetti et al. (2019), another study, always conducted in Italy, analysed through a residential cohort study the health effects of the population residing near a cement plant using NO_2_ dispersion models as a proxy for exposure to cement plant emissions [17]. The authors reported a higher mortality risk from cardiovascular diseases among women, particularly for cerebrovascular diseases, and no significant differences for respiratory diseases [17], in line with our results reporting such excesses even among men. As for hospitalization, our results are in agreement with those of the study by Ferroni et al. (2021) only with regard to diseases of the circulatory system but not for the sub-causes (for which we observed excesses) and for respiratory diseases.

EPIVenafro + 7 study presents strengths and limitations. The strength of the EpiVenafro + 7 study is the residential cohort design, one of the most advanced approaches adopted in other national environmental epidemiology studies [38–40]. This kind of study allows to examine different outcomes for the same type of exposure and data at an individual level makes it possible to follow each subject over time through the reconstruction of the residential history and to associate the exposure to the subject based on the residence duration at his/her address. Unlike most studies conducted for exposures to WTE/cement plants that used distance from residence to assess subject exposure, in the EPIVenafro + 7 study was constructed a dispersion model of a substitute for persistent pollution in the area under study, considering other sources that insist on the territory, such as the roads which, particularly in Venafro, generate an important vehicle traffic situation. Furthermore, assigning exposure to each subject through a dispersion model reduces the arbitrariness of subjective choice. It should also be emphasized that in the EPIVenafro + 7 study the georeferencing reached 100% and this allowed for no distortions due to the lack of subjects that can be distributed in the territory in a different way. The loss of 5% of the subjects coming from the mortality register and of 10% of the subjects coming from the HDF allowed us to be quite confident regarding the reliability of the estimates obtained since, in cohort studies a loss of 10% is considered physiological. In this way the EPIVenafro + 7 study meets what has been suggested by the recent scientific literature, which recommends both the use of dispersion models and geocoding for a more correct exposure assessment [26, 33]. The EPIVenafro + 7 study was also characterized by some limitations and a-priori study design. The size of the sample which in some cases generated an indicator estimate with low precision or even was not reported for reasons of privacy of the subjects because it was less than 3 cases. Despite the exhaust plumes emitted by the plants under examination contain a complex mixture of gaseous and particulate substances, they share very similar dispersion fate within the spatial study domain; the dispersion maps of a single substance may be used as an indirect measure (proxy) of such a complex exposure pattern. In this study NO_x_ have been chosen as a proxy of exposure to the plant plumes. It was dictated by two reasons: NO_x_ showed a sufficient spatial variability for an effective classification between less and more exposed [17, 41, 42]; stack measurement of other substances such as dust and SO_2_ showed poor quality or frequency in the reference year. As previously specified, the choice of 2016 as the reference year was due: (i) to the availability of hourly emission data from the two plants; (ii) the absence or reduced significant meteorological anomalies of the year, also reported at national level, since it must represent a typical weather year for a longer period. Therefore, the use of a dispersion model based on a single year considered representative of the follow-up period could represent a limit just in case of changes in the yearly averages in the follow-up period. However, since the meteorological characteristics and the emission data did not show significant changes over the study period, as reported in more detail in the final report available on line at  https://www.comune.venafro.is.it/ente/avvisi/137, it was assumed that the exposure model for the cohort did not undergo such alterations. Furthermore, while on the one hand the yearly average NO_x_ concentration was not representative of the daily concentration peaks, on the other hand our study aimed to define a medium-long term exposure; we therefore considered an adequate approximation to use the average NO_x_ concentration of the reference year as an exposure indicator. Another limitation regarded the fact that the attribution of complex exposure such as the multi-source one was carried out based only on the subject’s residential address. In fact, the residence may not adequately represent the real exposure of the subjects, as people spend several hours outside their home for personal and/or work reasons. The EPIVenafro + 7 study did not consider information relating to the movements of the cohort subjects during the day. This can lead to a miss-classification between residents in exposed and less-exposed areas and to the generation of biases which, however, usually lead to underestimating the risks since such miss-classification is non-differential. It is also important to highlight that the population near industrial areas shares multiple exposures and the methodology that leads to the identification of the isolated effect deriving from different polluting sources that insist on the same area is complex and sometimes not applicable. In particular, for exposure to pollutants generated from sources close to each other, it is not possible to uniquely attribute the effects of one source over another. Furthermore, as data were not available, confounders such as cigarette smoking, alcohol abuse, obesity, diet, physical activity, occupational exposure and other environmental exposure (home heating and other industries) were not directly considered. For these factors, even if it was reasonable to assume that there was no difference between residents in areas with different levels of exposure, a confounding effect cannot be excluded. On the other hand, since many of the personal habits listed above were recognized to be associated with socio-economic status, we can assume that the correction made in the analyses for the socio-economic DI may have considered, at least partially, individual variables and lifestyles not measured directly in the EPIVenafro + 7 study. In fact, the socio-economic deprivation index available at the census section level was used as a proxy for individual deprivation in order to adjust health risk estimates. This choice, in line with that of other studies of national relevance, was made with the awareness that this index is often associated with both environmental exposure and various causes of mortality/morbidity and that it could be affected by ecological bias.

## Conclusions

Concluding, the retrospective residential cohort study EPIVenafro + 7, for exposure to the industrial proxy NO_x_ highlights mortality/morbidity risk excesses for diseases of the circulatory system among subjects in class 3 and for some subgroups of cardiovascular and respiratory diseases among those most exposed, even if of low entity. The results for such exposure provide some signals regarding exposure to pollutants emitted in the study area and the onset of chronic diseases even if accompanied by uncertainty. The scientific evidence, especially as regards the health effects deriving from exposure to WTE plants, is inadequate. Even if of a weak entity, the results of the EPIVenafro + 7 study nevertheless represent a contribution on this topic which will need to be investigated. The extent of the excesses found highlight a situation that provides a series of signals worthy of further study including lifestyles and other individual risk factors is highlighted. Although the cohort study is one of the most advanced study designs, the uncertainty of the estimates can be reduced both by refining this methodology, improving accuracy and precision of the exposure, and by using other analytical investigation methodologies that epidemiology makes available and which could represent strategies to be used in the future to investigate the excesses of risk highlighted by the EPIVenafro + 7 study. In particular, in the area were recommended in-depth studies through cohort studies considering the use of occupational exposure matrices, the birth assistance certificates, the Cancer Registry, in-depth analysis of the results on population subgroups and a sample study on cardiovascular and respiratory diseases with questionnaires and human biomonitoring.

In conclusion, although the aforementioned in-depth analyses are recommended in order to reduce the uncertainty of the estimates, considering also that last year the World Health Organization published the new Air Quality Guidelines, it is now necessary to pursue environmental policies aimed at reducing concentrations of atmospheric pollutants in order to reduce the health burden deriving from exposure to air pollution and, although the new Air Quality Guidelines are not legally binding, it is desirable that they constitute the reference point for policies in all countries and in all sectors.

## Data Availability

The data that support the findings of this study are available from the Municipality of Venafro (data controller) but restrictions apply to the availability of these data, which were used under license for the current study, and so are not publicly available. Data are however available from the authors upon reasonable request and with permission of Municipality of Venafro.
